# PPARγ is reduced in the airways of non-CF bronchiectasis subjects and is inversely correlated with the presence of *Pseudomonas aeruginosa*

**DOI:** 10.1371/journal.pone.0202296

**Published:** 2018-08-16

**Authors:** Lucy D. Burr, Geraint B. Rogers, Alice C-H Chen, Steven L. Taylor, Simon D. Bowler, Rebecca L. Keating, Megan L. Martin, Sumaira Z. Hasnain, Michael A. McGuckin

**Affiliations:** 1 Immunity, Infection and Inflammation Program, Mater Research—University of Queensland, Translational Research Institute, Wooloongabba, QLD, Australia; 2 Department of Respiratory Medicine, Mater Misericordiae Brisbane Ltd, South Brisbane, QLD, Australia; 3 SAHMRI Infection and Immunity Theme, School of Medicine, Flinders University, Adelaide, Australia; Lee Kong Chian School of Medicine, SINGAPORE

## Abstract

**Background:**

Chronic airway inflammation in conditions such as cystic fibrosis (CF) and non-CF bronchiectasis is characterised by a predominant neutrophilic inflammatory response, commonly due to the presence of pathogenic bacteria such as *Pseudomonas aeruginosa*. We hypothesised that down-regulation of the anti-inflammatory nuclear transcription regulator peroxisome proliferator-activated receptor gamma (*PPAR*γ in non-CF bronchiectasis subjects may explain why this exuberant neutrophilic inflammation is able to persist unchecked in the inflamed airway.

**Methods:**

PPARγ gene expression was assessed in bronchoalveolar lavage fluid (BAL) of 35 macrolide naïve non-CF bronchiectasis subjects and compared with that in 20 healthy controls. Human RNA was extracted from pelleted BAL and PPARγ expression was determined by reverse-transcription quantitative PCR. Bacterial DNA was extracted from paired induced sputum and total bacterial load was determined by 16S rRNA qPCR. Quantification of individual bacterial species was achieved by qPCR.

**Results:**

PPARγ expression was lower in subjects with non-CF bronchiectasis compared with healthy control subjects (control: 1.00, IQR 0.55–1.44, n = 20 vs. Bronchiectasis: 0.49, IQR 0.12–0.89; n = 35; p<0.001, Mann-Whitney U test). This lower PPARγ expression correlated negatively with *Pseudomonas aeruginosa* (r = -0.53, n = 31; p = 0.002). No significant association was seen between PPARγ and total bacterial levels or levels *Haemophilus influenzae*.

**Conclusion:**

PPARγ is expressed in low levels in the airways of non-CF bronchiectasis subjects, despite an aggressive inflammatory response. This low level *PPAR*γ expression is particularly associated with the presence of high levels of *P*. *aeruginosa*, and may represent an intrinsic link with this bacterial pathogen.

## Introduction

Non-cystic fibrosis bronchiectasis is a chronic pulmonary condition characterised by exuberant airway inflammation and damage caused by chronic airway infection and exacerbation [1]. Infection with the opportunistic pathogen *Pseudomonas aeruginosa* is associated with accelerated lung function decline [2] and increased mortality [3,4]. Why *P*. *aeruginosa* colonised patients have poorer outcomes is unclear, but is likely to reflect an imbalance between the pro-inflammatory and anti-inflammatory nature of the host-pathogen relationship.

In cystic fibrosis (CF), the expression of anti-inflammatory nuclear transcription regulator peroxisome proliferator-activated receptor gamma (*PPAR*γ is low compared to healthy controls, and appears to be reduced further in the presence of *P*. *aeruginosa* [5]. *PPAR*γ binds to exogenous lipid-like ligands and then interacts with specific PPAR response elements on nuclear DNA to modulate gene expression [6]. Inhibition of *PPAR*γ has been shown to result in a pro-inflammatory phenotype by interfering with its inhibitory action on the target genes of NFκB [6], supportive of the overall anti-inflammatory effect of *PPAR*γ. The mechanism by which *PPAR*γ production is suppressed in CF is not known. However, *in vitro* studies have demonstrated that *P*. *aeruginosa* acyl-homoserine lactones (AHLs)–lipid-like exoproducts of *P*. *aeruginosa*—suppress expression of *PPAR*γ in various cell types, including epithelial and endothelial cells [5,7,8]. Bacterial AHLs have been shown to enter, and function in, mammalian cells [9–11]. One way that *P*. *aeruginosa* might interfere with the host immune response is through the suppression of PPARγ function, through inhibition of *PPAR*γ transcription or translation. Despite the availability of commercial PPARγ agonists, which might provide clinical benefit, there have been no reported investigations of the role of *PPAR*γ in non-CF bronchiectasis.

We examined the expression of *PPAR*γ in the airways of macrolide naïve non-CF bronchiectasis patients who were recruited as part of a prospective randomised controlled trial [12]. Further, we explored the relationship between *PPAR*γ expression and the presence of important colonising pathogens, such as *P*. *aeruginosa*.

## Methods

### Study population

The study was approved by the Mater Human Research Ethics Committee and all participants provided written informed consent. Subjects in this study were participants in the BLESS trial [12]. A subgroup of 41 participants underwent bronchoscopic sampling at baseline, along with 20 healthy controls. BLESS participants were recruited for this subgroup sequentially at the same time as they were recruited to the parent study and were only excluded if they had a co-morbidity that would preclude safe bronchoscopy and biopsy, or after the pre-specified number of participants had been reached. The current analysis is based upon the 35 bronchoscopy subjects and 20 healthy controls from whom sufficient bronchoalveolar lavage (BAL) sample was obtained for gene expression analysis. Table 1 shows the baseline clinical characteristics of these two groups. Six subjects in the non-CF bronchiectasis group did not have sufficient BAL sample for analysis, however, there was no significant difference between this group and the original 41 patients recruited for bronchoscopy in the trial (data not shown).

**Table 1:** Baseline characteristics of the 35 bronchiectasis subjects and 20 healthy control subjects included in this analysis.

	Bronchiectasis (n = 35)	Control (n = 20)
Age, mean (SD), y	62.8 (6.6)	35.8 (11.8)
Female sex, No. (%)	25 (71.4)	12 (60.0)
FEV1 (post bronchodilator) % (SD)	75.8 (12.5)	100.2 (12.0)
*P*. *aeruginosa* PCR positive, no. (%)	31 (88.6)	0 (0)
*H*. *influenzae* PCR positive, no (%)	13 (37.1)	0 (0)
No Exacerbations in prior year, mean (SD)	1.9 (1.7)	0 (0)
Ex-smokers, No. (%)	4 (11.4)	0 (0)
Pack Years, mean (SD)	0.2 (0.6)	0 (0)
Sputum weight g, mean (SD)	16.2 (10.1)	N/A

### BAL and sputum processing

Gauze-filtered BAL was separated into sterile specimen jars and transported to the laboratory on ice. One portion was sent for cell counting. BAL was then further divided into 1 mL aliquots. A minimum of 2 aliquots were centrifuged at 500 *x g* for 5 min at 4°C to pellet large cellular debris. The pellet was then frozen rapidly and stored at -80°C prior to RNA extraction. Matched, induced sputum was frozen rapidly and stored at -80°C until subsequent molecular analysis.

### Cell counts

BAL was processed within 60 minutes of collection. After filtration, the sample was centrifuged, the supernatant aspirated and the cell pellet resuspended in PBS for cell counting in the presence of trypan blue and both Blue and Red Rapi-Diff cell stains.

### DNA extraction

DNA extraction for both PCR and microbiome analysis was performed on 200 μL portions of paired induced sputum using a combination of physical disruption and a phenol/chloroform-based methodology as described previously [13].

### RNA extraction and cDNA synthesis

RNA extraction from BAL was performed using an on-column method as per the manufacturer’s instructions for RNeasy RNA Extraction Kit (Qiagen, Netherlands). cDNA synthesis for the non-amplified RNA samples was performed using the iScript cDNA synthesis kit (Bio-Rad, California USA), as per the manufacturer’s instructions.

### PPARγ gene expression by RT-PCR

*PPAR*γ gene expression was measured using quantitative reverse-transcription PCR (qRT-PCR). The total reaction volume was 7.5 μL with the following components: Sybr 3.75 μL, primers (forward and reverse) 0.75 μL of 200 nM, ROX 0.15 μL, H_2_O 0.35 μL, Template cDNA 2.5 μL. Thermal cycling conditions were as follows: Hold 95°C 2min, Cycle (x40), 95°C 30 s, 60°C 30 s, 72°C 30 s, Hold 72°C 2 min. Each reaction was performed in duplicate. Gene expression calculated by the 2ˆ(–delta delta CT) method, normalized to the expression of the housekeeping gene, β-actin, which was measured using cycling conditions: Hold 95°C 2min, Cycle (x40), 95°C 15 s, 59°C 45 s, 72°C 20 s, Hold 72°C 20 s. All products underwent melt curve analysis.

Primer sequences were as follows (optimized from Griffin *et al* 2012 [5]):

PPARγforward 5'-AGCTGAACCACCCTGAGTCC-3'
PPARγ reverse 5'-TCATGTCTGTCTCCGTCTTCTTG-3'
β-actin forward 5'-GGCTGGCCGGGACCTGACTGA-3'
β-actin reverse 5'-CTTCTCCTTAATGTCACGCACG-3'

### Quantitative PCR for the determination of bacterial load

Total bacterial load was determined in paired sputum samples using a Taqman assay directed against a conserved region of the 16S rRNA gene, as described previously [14]. *P*. *aeruginosa* levels were determined using a Taqman assay, in which a 117 bp region between positions 330 to 447 of the *P*. *aeruginosa oprL* gene was amplified, as described previously [13]. *H*. *influenzae* density was determined using a Taqman assay, in which a 90-bp region between positions 518 to 608 of the *H*. *influenzae hel* gene was amplified [13].

### Clinical correlates

Lung function and assessment of clinical measures were performed at enrolment as described previously [12]. Spirometry was performed pre- and post- inhalation of salbutamol bronchodilator. Serum CRP was measured at each visit. A 24-hour sputum sample and a spontaneously expectorated sputum sample were collected at each visit for volume quantification, microbiology, and differential cell count.

## Results

**Fig 1:**
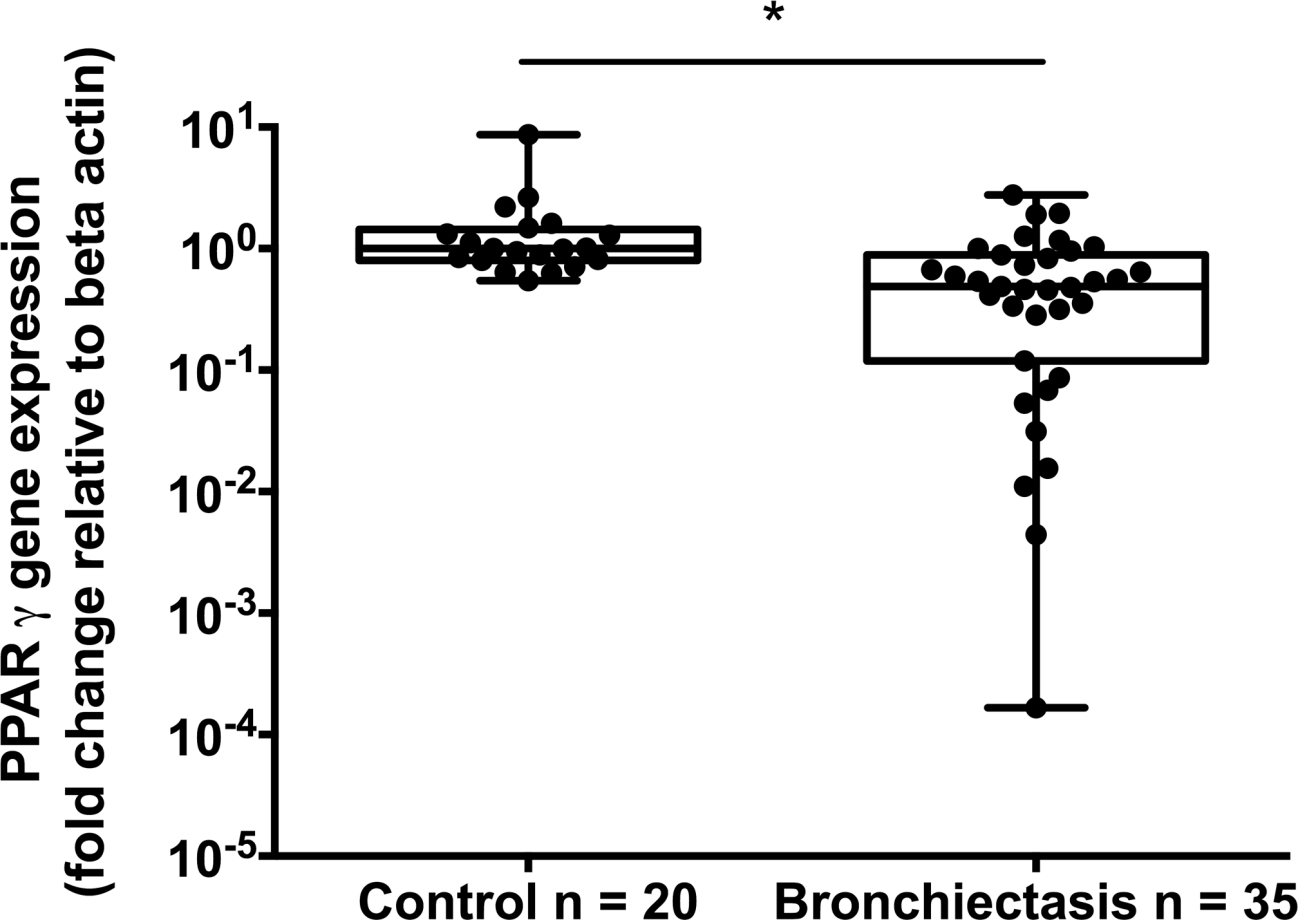
Relative PPARγ gene expression, as determined by RT-PCR in BAL-derived cells from healthy control volunteers and macrolide naïve non-CF bronchiectasis subjects. Gene expression levels were expressed relative to the expression of the housekeeping gene, β actin and normalised to the median of the control group. Points represent individual subjects. Significance determined by Mann-Whitney U test. Significant values denoted by an asterisk (*).

**Fig 2:**
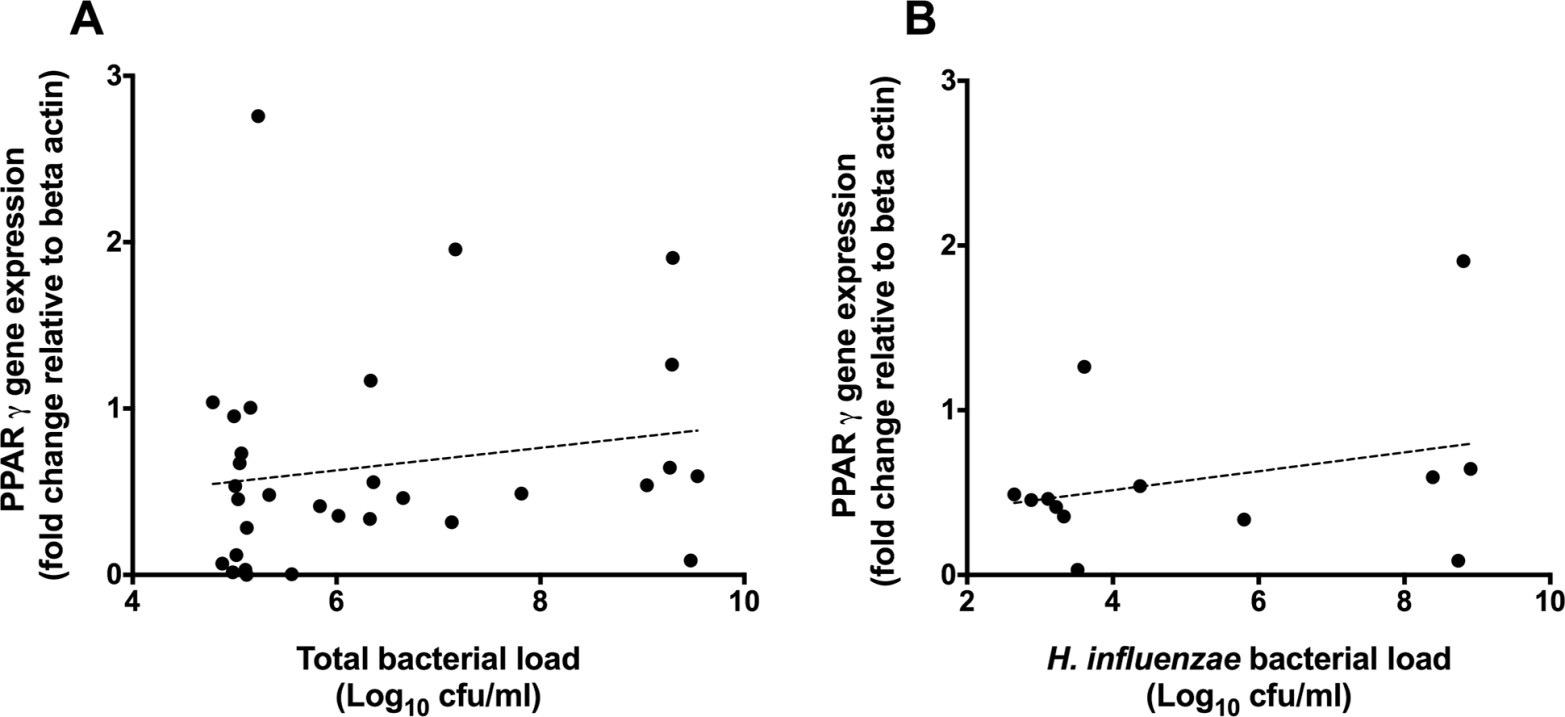
**PPARγ gene expression levels (y axis) correlated with total bacterial load (panel A (r = 0.24, p = 0.194)) and *H*. *influenza* (panel B (r = 0.30, p = 0.325)), as determined by species specific PCR (x axis).** Points represent individual patient values, dotted line represents the line of best fit.

**Fig 3:**
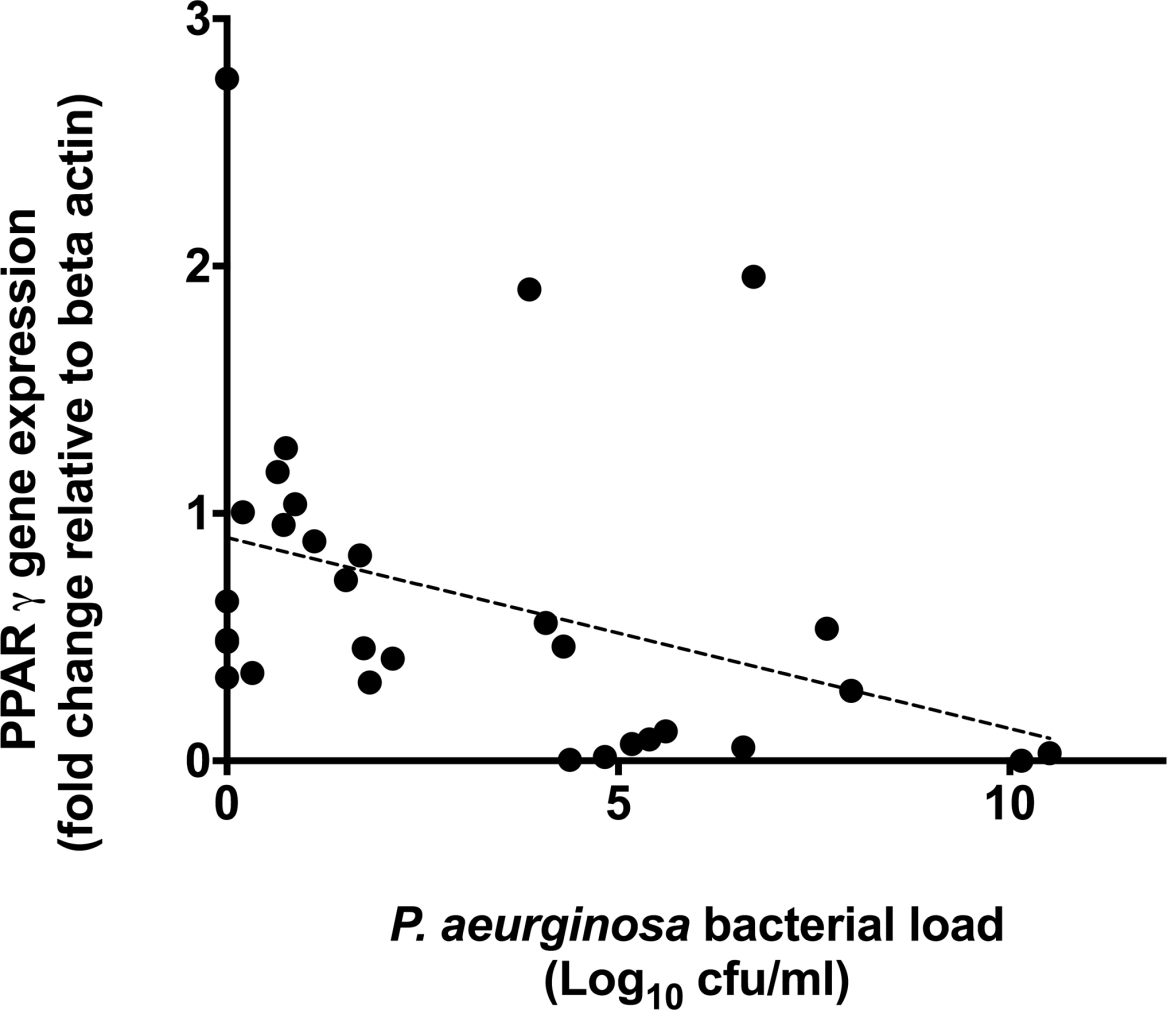
PPARγ gene expression levels (y axis) correlated with *P*. *aeruginosa* bacterial load (x axis) as determined by PCR. There was a significant negative correlation of PPARγ gene expression with *P*. *aeruginosa* (r = -0.53, p = 0.002). Points represent individual patient values, dotted line represents the line of best fit.

Subject baseline respiratory characteristics are described in Table 1. *PPAR*γ gene expression was significantly lower in the airways of non-CF bronchiectasis subjects compared with healthy controls (control: 1.00, IQR 0.55–1.44, n = 20 vs. Bronchiectasis: 0.49, IQR 0.12–0.89; n = 35; p<0.001, Mann-Whitney U test), Fig 1. Within patients with bronchiectasis, *PPAR*γ expression did not correlate with either total bacterial load (as measured by 16S qPCR; r = 0.24, p = 0.194) or *H*. *influenzae* bacterial load (r = 0.30, p = 0.325) (Fig 2). However, *PPAR*γ expression was negatively correlated with total *P*. *aeruginosa* bacterial load (r = -0.53, n = 31; p = 0.002; Fig 3).

**Fig 4:**
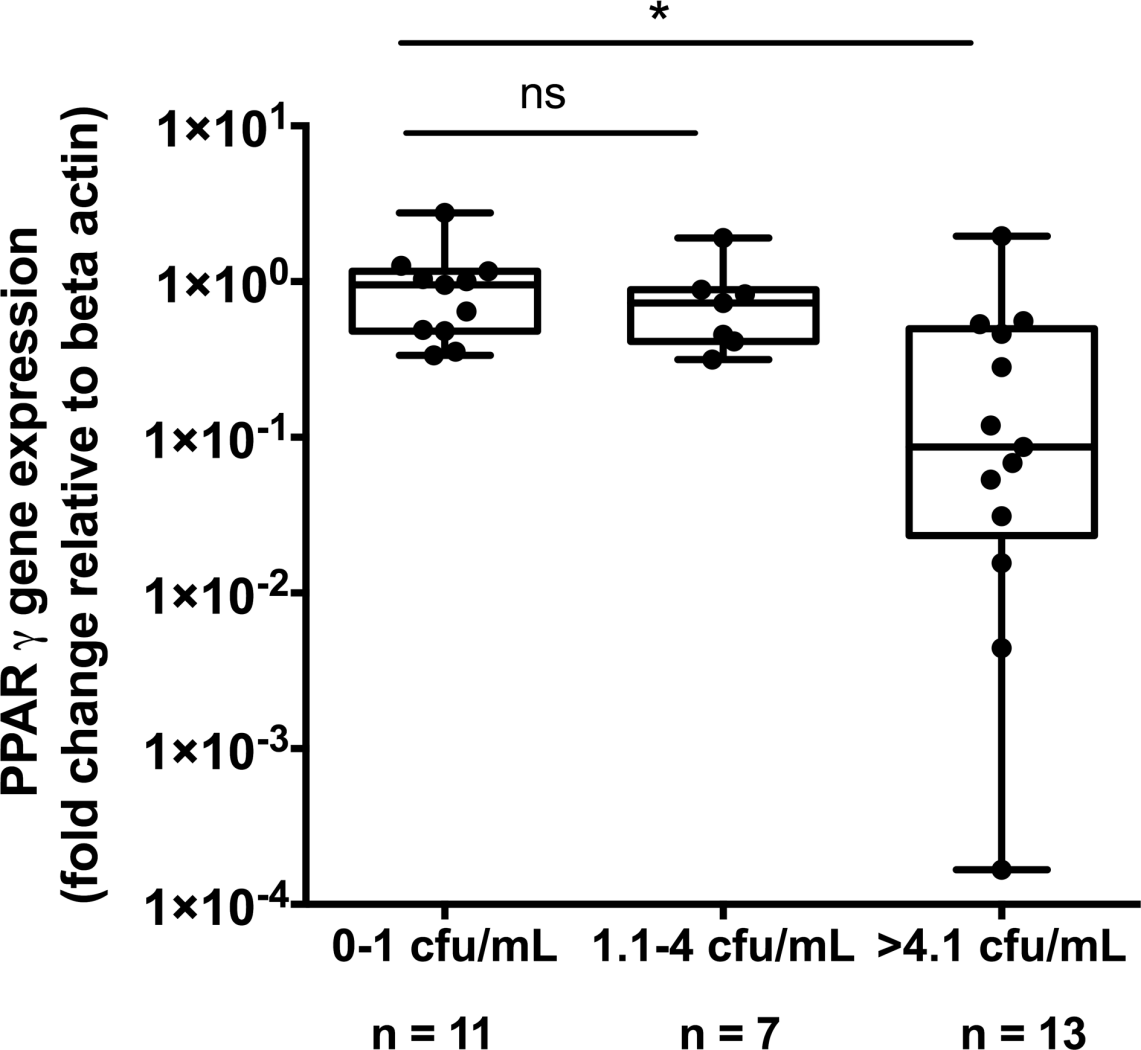
PPARγ gene expression levels in BAL-derived cells (y axis) divided into no, low and high *P*. *aeruginosa* bacterial load, as determined by PCR. There is a significant reduction in PPARγ gene expression with high levels of *P*. *aeruginosa* colonisation. Points represent individual subject values. Significance determined by Mann-Whitney U test. Significant values denoted by an asterisk (*).

To further investigate the relationship between *PPAR*γ and *P*. *aeruginosa*, *PPAR*γ expression was assessed relative to *P*. *aeruginosa*. Bacterial load was divided into 3 groups; <1 log CFU/mL (essentially representing absence of *P*. *aeruginosa*); 1.1–4 log CFU/mL (representing low level colonisation with *P*. *aeruginosa*) and >4.1 log CFU/mL (representing high level *P*. *aeruginosa* colonisation). *PPAR*γ was significantly lower in patients with high levels of *P*. *aeruginosa* colonisation compared with no or low *P*. *aeruginosa* (low 0.953 (IQR 0.336–1.17) n = 11 vs High 0.087 (IQR 0.023–0.498) n = 13; p = 0.002, Mann-Whitney U test) (Fig 4).

**Fig 5:**
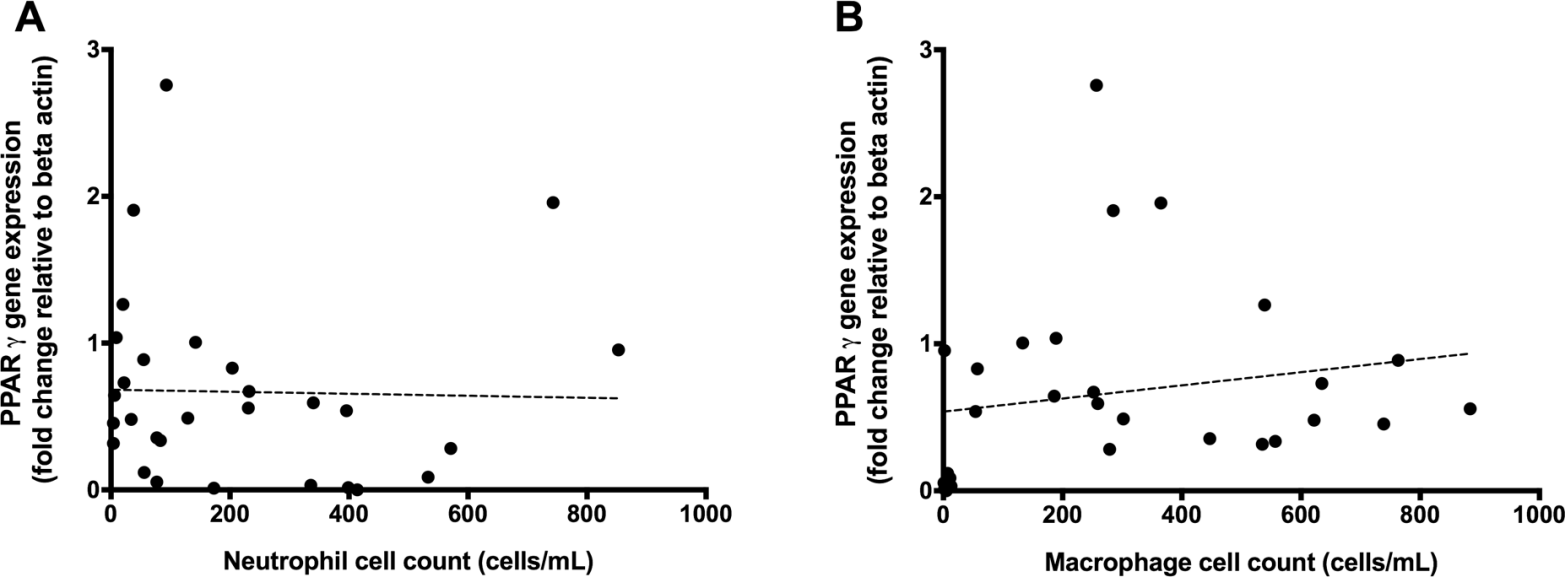
Correlation between total neutrophil count in induced sputum against relative PPARγ gene expression in BAL-derived cells (panel A (r = -0.19, p = 0.329)). This is compared with the correlation between total macrophage count in induced sputum with relative PPARγ gene expression in BAL-derived cells (panel B (r = 0.38, p = 0.044)). Points represent individual patient values, dotted line represents the line of best fit.

**Fig 6:**
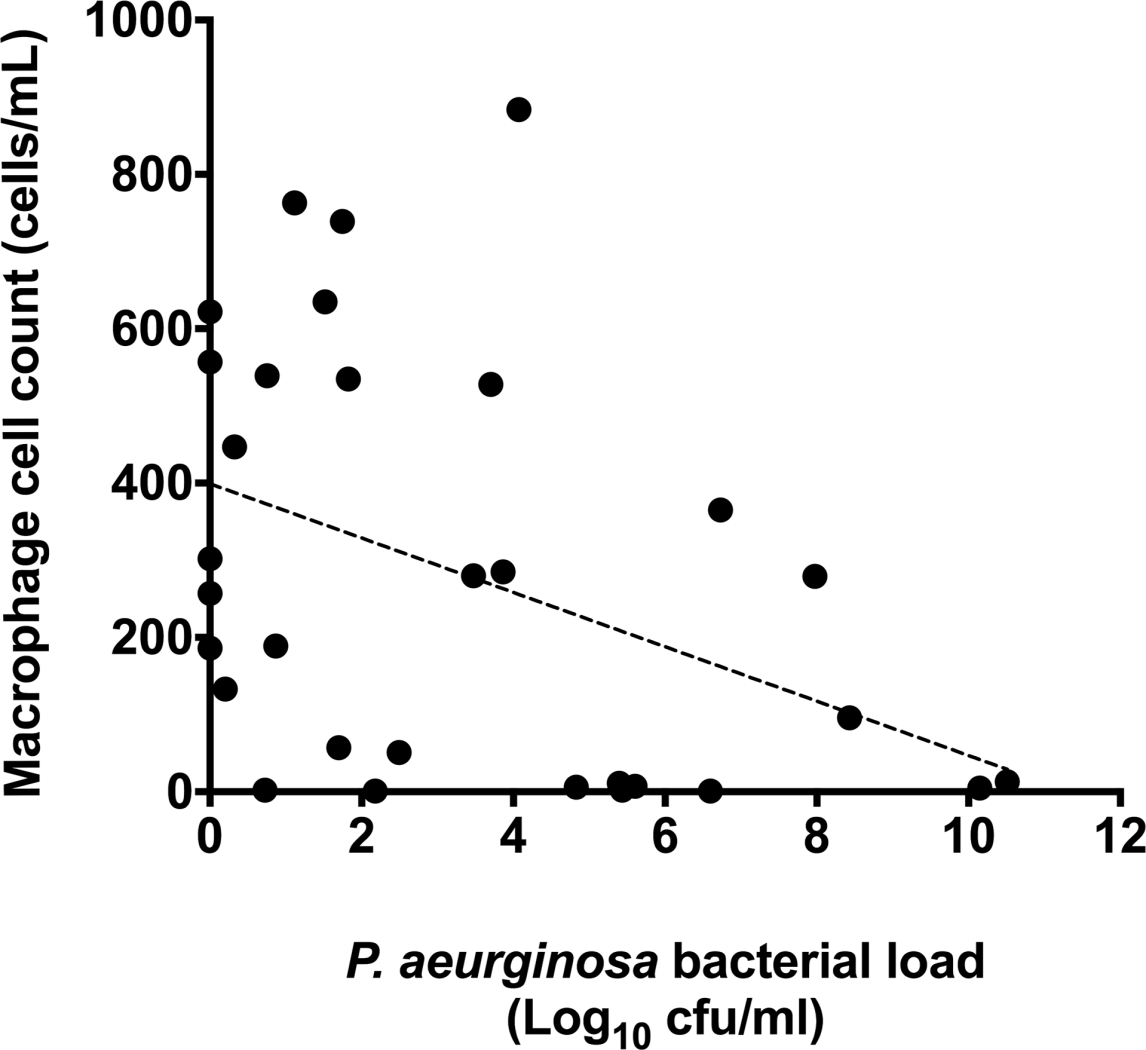
Correlation between total macrophage count against total *P*. *aeruginosa* bacterial load (r = -0.408, p = 0.023). Points represent individual patient values, dotted line represents the line of best fit.

Levels of PPARγ gene expression were assessed relative to airway immune cell counts. PPARγ gene expression was correlated to total neutrophil and macrophage levels in BAL fluid. No significant correlation was observed between total neutrophil count and PPARγ gene expression (r = -0.19, p = 0.329) (Fig 5A). However a weak, but significant positive correlation was observed between PPARγ gene expression and total macrophage count (r = 0.38, p = 0.044) (Fig 5B). *P*. *aeruginosa* load was then correlated with total macrophage levels, where there was a significant, negative correlation between macrophages and *P*. *aeruginosa* (r = -0.408, p = 0.023) (Fig 6).

**Fig 7:**
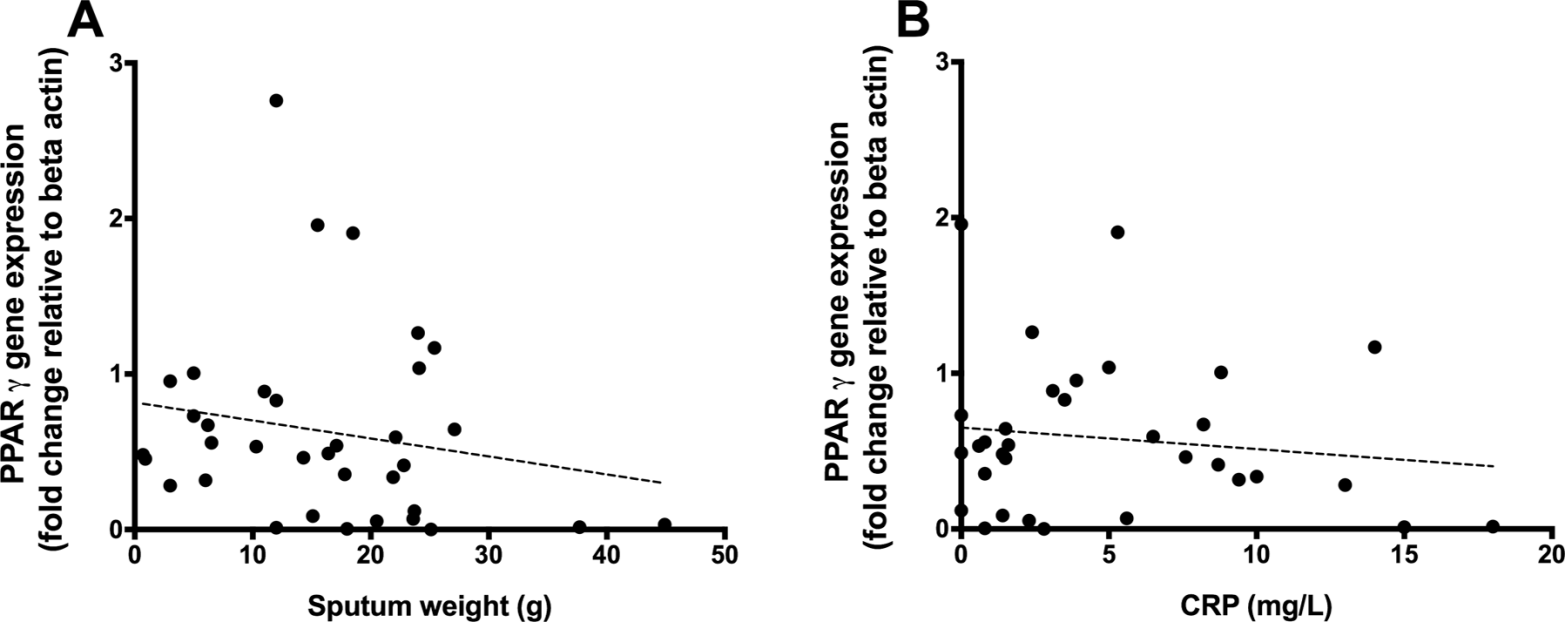
Correlation between PPARγ relative gene expression in BAL-derived cells against daily sputum production in non-CF bronchiectasis subjects (panel A (r = -0.190, p = 0.276)). This is compared with the correlation between PPARγ relative gene expression in BAL-derived cells against CRP in non-CF bronchiectasis subjects (panel B (r = -0.137, p = 0.449)). Points represent individual patient values; dotted line represents the line of linear regression.

Agonising PPARγ has been implicated in the inhibition of stimulated mucin expression, potentially reducing sputum volume in subjects with excessive inflammation [15]. To investigate whether airway *PPAR*γ gene expression was related to sputum amount, levels were correlated with 24 hour sputum weight. There was a trend to lower *PPAR*γ gene expression with increasing sputum volume, suggesting that the more *PPAR*γ expression, the less sputum produced. However, this relationship did not achieve statistical significance (r = -0.190, p = 0.276) (Fig 7A). This was also reflected with measurements of CRP (r = -0.137, p = 0.449). However, again, the association was weak and non significant (Fig 7B).

To determine whether the alterations in *PPAR*γ were associated with important clinical outcomes, correlations with lung function (as measured by FEV1% predicted) were performed. There was no significant correlation between PPARγ expression and lung function as measured by FEV1 (r = 0.082, p = 0.225).

## Discussion

Our aim was to assess the relevance of PPARγ in the context of *P*. *aeruginosa* colonisation in non-CF bronchiectasis. PPARγ is an anti-inflammatory product of leukocytes, which has been shown to inhibit, or switch off, NFκB in response to lipids [6]. In doing so, PPARγ might help to limit exuberant inflammation within the context of suppurative lung disease. *P*. *aeruginosa* AHLs are lipid-like structures that readily interact with PPARγ. *P*. *aeruginosa* AHLs have been shown to reduce PPARγ gene expression in endothelial and other cell lines when exposed *in vitro* [8].

Low levels of PPARγ expression have been reported in CF airways and that PPARγ levels are lowest in patients colonised by *P*. *aeruginosa* [5]. Our data suggests reduced levels of *PPAR*γ are also characteristic of the non-CF bronchiectasis airways, despite an aggressive inflammatory response. In keeping with the observations made in patients with CF, high airway levels of *P*. *aeruginosa* in patients with bronchiectasis are associated with low levels of *PPAR*γ expression, suggesting an intrinsic link with this bacterial species. This is supported by data demonstrating that agonising PPARγ inhibits *P*. *aeruginosa* biofilm production *in vitro* [16], and is further supported by evidence suggesting that clearance of macrophages infected with *P*. *aeruginosa* is enhanced with activation of PPARγ[17]. Whilst further work would be necessary to confirm these findings, our data supports a specific bacterial interaction between *P*. *aeruginosa* and PPARγ.

We assessed PPARγ gene expression in cells derived from the airway fluid of healthy and non-CF bronchiectasis subjects. These samples comprise mainly immune cells, such as neutrophils, eosinophils and macrophages, with minimal contribution of epithelial cells. Airway measures of *PPAR*γ therefore likely originate from luminal immune cells in this context. In order to determine whether there was any correlation with *PPAR*γ expression and airway immune cells, *PPAR*γ expression was correlated with absolute and percentage immune cell counts. Interestingly there was no correlation with airway neutrophils, but there was a weak but significantly positive correlation with airway macrophages. This suggests that macrophages may be the primary source of PPARγin non-CF bronchiectasis airways. Interestingly, the more *P*. *aeruginosa* present in the airways, the fewer macrophages were found. There is evidence that the *P*. *aeruginosa* AHL molecule 3-oxo-12-HSL accelerates apoptosis in macrophages [18,19], suggesting that, in the presence of *P*. *aeruginosa*, there is the potential for fewer, less active macrophages and less PPARγ.

In order to determine whether *PPAR*γ levels could predict important clinical outcomes, *PPAR*γ, measured at baseline, was correlated with clinical outcomes. There were no significant correlations with specific clinical outcomes, however, there was a trend to decreased sputum amount with increasing *PPAR*γ expression. This trend is consistent with previous literature implicating *PPAR*γ agonism with decreased mucus hypersecretion [15,20].

Our study had a number of limitations. Firstly, we focused on levels of *PPAR*γ gene expression, rather than on its functional activity. However, previous studies have shown a strong correlation between *PPAR*γ gene expression and protein activity suggesting gene expression is an accurate reflection of activity [21]. Previous studies assessing airway *PPAR*γ expression in association with disease states have also arrived at similar conclusions [5,22]. Another limitation was that the healthy control group was significantly younger than the diseased group, which can affect PPARγ [23] and could explain the differences between the groups. The healthy group, by its nature, did not have any airway disease, meaning that their lung function was better, they did not have colonising pathogenic airway bacteria, and had no evidence of systemic inflammation, all of which necessarily confounded the analysis. While we can say that *PPAR*γ expression is lower in the diseased group compared with a non-diseased group, the specific reason for that difference cannot be reliably answered from the available data. Within the diseased group there were significant correlations between *PPAR*γ expression and specific disease markers such as presence of *P*. *aeruginosa* and abundance of macrophages. Correlation is not causation however. It must be also noted that in the disease cohort used in this study, there was a high prevalence of patients colonised by *P*. *aeruginosa* (as detected by qPCR). This rate is higher than previously found in the non-CF bronchiectasis population, [12] therefore further studies characterising *P*. *aeruginosa* levels in other non-CF bronchiectasis cohorts are required.

Nonetheless, the data presented here demonstrates for the first time that lower levels of *PPAR*γ are present in the airways of non-CF bronchiectasis subjects, despite an aggressive inflammatory response. This low level *PPAR*γ expression is particularly associated with the presence of *P*. *aeruginosa*, hinting at a possible mechanism involving this bacterial species. This is similar to data acquired from patients with CF [5] and supports the notion that low levels of traditional anti-inflammatory molecules may also play a role in disease due to the inability of the host to rein in exuberant inflammation. Notably, PPARγ agonists are commercially available treatments for Type 2 Diabetes Mellitus and may offer a potential therapeutic agent to aid in the treatment of inflammatory airways diseases.
